# Medical students’ perception of simulation-based assessment in emergency and paediatric medicine: a focus group study

**DOI:** 10.1186/s12909-021-02957-5

**Published:** 2021-11-19

**Authors:** Anne-Laure Philippon, Jennifer Truchot, Nathalie De Suremain, Marie-Christine Renaud, Arnaud Petit, Georges-Louis Baron, Yonathan Freund

**Affiliations:** 1 Emergency Department, Hôpital Pitié-Salpêtrière, Assistance Publique-Hôpitaux de Paris, 83, bd de l’hôpital, 75013 Paris, France; 2 Sorbonne Université, Paris, France; 3 Department of Learning Sciences, EDA Laboratory, Université Sorbonne Paris Cité, Paris, France; 4 Emergency Department, SMUR, Hôpital Cochin, Assistance Publique – Hôpitaux de Paris (APHP), Paris, France; 5 Emergency Department, Trousseau Hospital, Assistance Publique – Hôpitaux de Paris (APHP), Paris, France; 6 Department of Pediatric Hematology-Oncology, Trousseau Hospital, Assistance Publique – Hôpitaux de Paris, Paris, France

**Keywords:** Education, Undergraduate medical, Assessment, Simulation in healthcare, Activity theory

## Abstract

**Background:**

Although simulation-based assessment (SBA) is being implemented in numerous medical education systems, it is still rarely used for undergraduate medical students in France. Objective structured clinical examinations (OSCEs) will be integrated into the national medical curriculum in 2021. In 2016 and 2017, we created a mannequin SBA to validate medical students’ technical and psychometric skills during their emergency medicine and paediatric placements. The aim of our study was to determine medical students’ perceptions of SBA.

**Methods:**

We followed the grounded theory framework to conduct a qualitative study. A total of 215 students participated in either a paediatric or an emergency medicine simulation-based course with a final assessment. Among the 215 participants, we randomly selected forty students to constitute the focus groups. In the end, 30 students were interviewed. Data were coded and analysed by two independent investigators within the activity theory framework.

**Results:**

The analyses found four consensual themes. First, the students perceived that success in the SBA provided them with self-confidence and willingness to participate in their hospital placements (1). They considered SBA to have high face validity (2), and they reported changes in their practice after its implementation (3). Nevertheless, they found that SBA did not help with their final high-stakes assessments (4). They discussed three other themes without reaching consensus: stress, equity, and the structure of SBA. After an analysis with activity theory, we found that students’ perceptions of SBA underlined the contradictions between two systems of training: hospital and medical. We hypothesise that a specific role and place for SBA should be defined between these two activity systems.

**Conclusion:**

The students perceived that SBA would increase self-confidence in their hospital placements and emphasise the general skills required in their future professional environment. However, they also reported that the assessment method might be biased and stressful. Our results concerning a preimplementation mannequin SBA and OSCE could provide valuable insight for new programme design and aid in improving existing programmes. Indeed, SBA seems to have a role and place between hospital placements and medical schools.

**Supplementary Information:**

The online version contains supplementary material available at 10.1186/s12909-021-02957-5.

## Background

Following the recommendations of the Accreditation Council for Graduate Medical Education, competency-based medical education (CBME) principles have been widely implemented in most medical education systems [1]. Therefore, student assessments must meet the requirements of a competency-based approach even though it remains a barrier to CBME development [2]. Competencies can feel abstract while being context dependent, resulting in difficulties in finding meaningful assessment tools [2, 3]. As underlined by Carraccio, assessment remains the “Achilles’ heel” of CBME, even though significant progress has been made in this field [3–5]. Consequently, medical education systems face the challenge of evaluating competencies through validated, high-stakes assessments, such as the already existing NBME licensing board assessments [4, 6–8].

To achieve high-quality assessment, multiple modalities can be used to assess competencies: direct observation, multisource feedback, and simulation [4, 9]. CBME creates an opportunity for the simulation community to participate in a competency-based assessment system, both as formative (assessment for learning) or summative (assessment of learning) assessments [10, 11]. However, simulation-based assessment (SBA) offers a semiauthentic, complex environment and the opportunity to practice a full range of clinical skills without exposing real patients to any risks. For those reasons, it appears to be a suitable tool in the field of emergency medicine (EM), where health care providers manage rare and critical conditions [8, 12, 13].

The use of an objective structured clinical examination (OSCE) or mannequin-based simulations for summative assessments has emerged for postgraduate practice and interprofessional training, and their feasibility and acceptability have been demonstrated [14–17]. However, it is still not routinely used for medical students in EM. Moreover, a recent Canadian study underlined the need for research on the role and the optimal way to incorporate high-stakes summative SBAs in EM training [18]. In France, OSCE or SBA uses remain unusual and varied, but in 2022, a new curriculum reform will implement an OSCE for medical students. When this research began, the students had never participated in either OSCE or mannequin-based simulation.

Thus, we developed two mannequin SBAs within the emergency medicine and paediatric curriculum of one medical school. However, although existing research addresses how to develop and use mannequin SBA, there remains a gap regarding learners’ perspectives [19, 20]. Learners’ reactions and perceptions of assessment could impact their engagement in the learning and assessment processes; therefore, this issue should be taken into consideration [18, 21, 22]. Therefore, we aimed to collect information on medical students’ perceptions of these new assessments.

## Methods

We conducted prospective qualitative research after validation of the protocol by the French Society of Intensive Care Medicine (SRLF) ethical committee (n°16–55). All focus group participants received written and oral information and signed an informed consent form. The study method reporting followed the COREQ framework, which is a 32-item checklist generated from a systematic literature review to help authors report on qualitative studies [23].

### Characteristics of the research team and reflexivity

The main investigator (ALP) has a master’s degree in learning sciences and previous experience with focus group interviews. YF, JT and ALP are graduated simulation trainers and attending physicians in the emergency department. MCR is an internist doctor working in a medical education department. AP and NdS are paediatricians and trainers in the paediatrics simulation curriculum. To enhance the credibility of the results, we worked with an outside expert, GLB, who is a learning sciences professor. Before designing the study, the main investigator analysed some bias linked to her representations of simulation-based training, assessment and emergency medicine. The aim of this process was to identify pitfalls in the field, such as assumptions and beliefs regarding SBA, and to acknowledge their potential influence. Because JT also analysed the data, she underwent the same process. The main investigator introduced herself as a learning sciences student and an emergency physician conducting a research project in the medical education domain.

### Study design and theoretical framework

To understand the medical students’ perception of SBA, the grounded theory approach was used to produce emergent themes and theories, as we did not have a “preconceived theory in mind” [24–26]. With this approach, the theories emerge from the data and could be analysed in regard to another theory. The focus group method was chosen to foster discussions between participants and generate point-counterpoint discussion [27].

### Setting: description of the simulation-based courses and assessments

The undergraduate medical curriculum lasts 6 years in France. At the end of the sixth year, undergraduate students undergo a national high-stakes assessment serving a classification purpose, which allows them to choose both a specialty and their residency university. During the final 3 years, medical students divide their time between hospital placements and faculty courses (including lectures, tutorials or simulation-based training). The simulation courses followed a traditional structure (prebriefing, briefing, scenario, debriefing).

The study took place in a single medical school affiliated with 4 teaching hospitals and 18 urban hospitals within Paris Sorbonne University in Paris, France.

#### Simulation course in the emergency medicine and intensive care medicine curriculum (EM-ICMC)

**Fig. 1:**
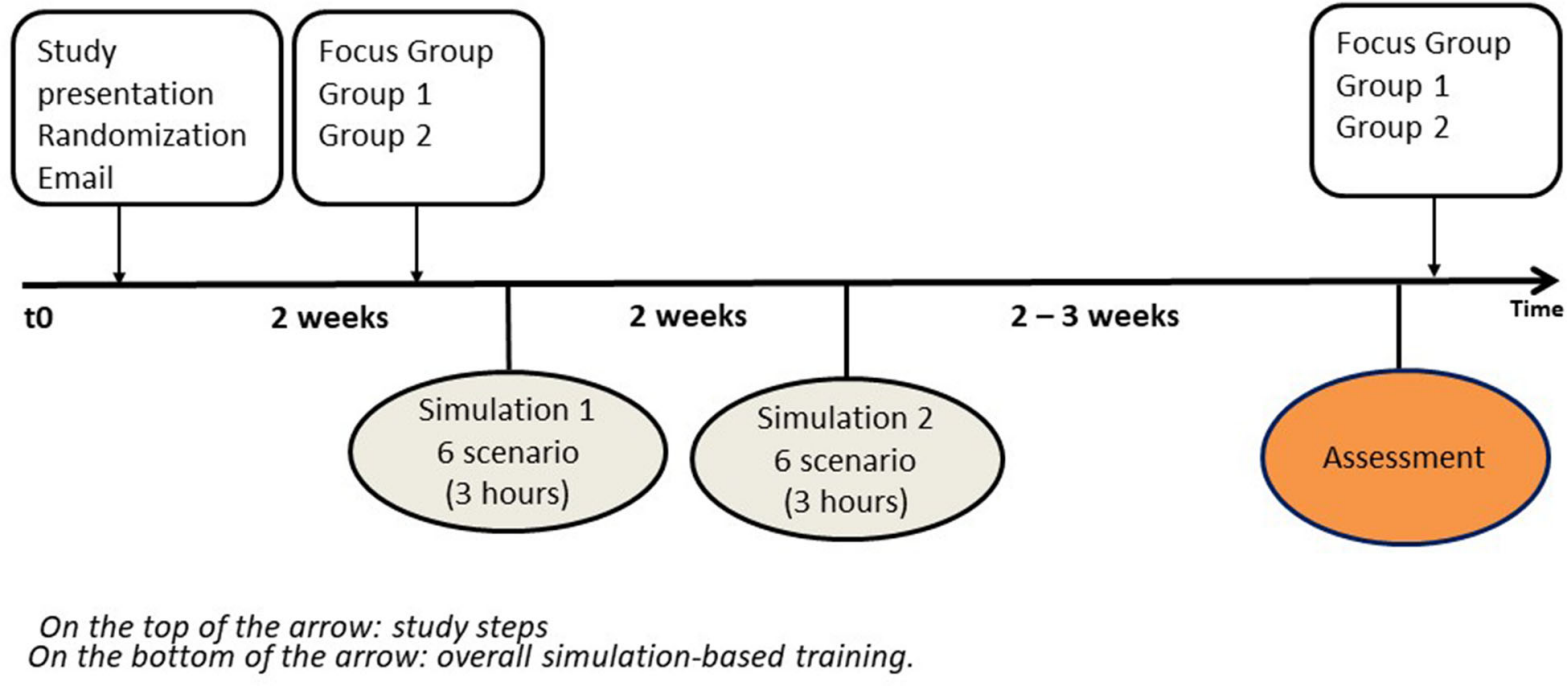
Simulation course and assessment within the Emergency Medicine – Intensive Care Medicine curriculum – 4th year medical students

Fourth-year medical students participated in two three-hour simulation-based courses. During each course, two or three students had to participate in one scenario lasting 8 to 10 min. The summative SBA took place during a third simulation session. The SBA started with a collective prebriefing, followed by medical students participating in pairs, in one EM scenario (Fig. 1). The debriefing took place in two stages: immediately after the scenario for the two “assessed” medical students and at the end of the “assessment session” with all the students. Assessors used specific assessment scores developed for each clinical case, as none existed to assess medical students in emergency medicine. The scores assessed medical students’ technical and nontechnical skills. Medical students had to complete two requirements to succeed: a grade higher than 10/20 and the completion of all the mandatory items (4 to 6 among 20 according to the scores).

#### Simulation-based assessment in the paediatric curriculum (PC) (Fig. 2)

Fifth-year medical students participated in a three-hour simulation-based course that included three sessions on paediatric basic and advanced life support, followed by an individual SBA of a paediatrics basic-life support clinical case. The SBA took place immediately after the end of the simulation-based courses. A single assessor assessed each student’s paediatric basic life support performance using a score derived from the ILCOR guidelines (Additional file 3: Annex 1, [28]).

**Fig. 2:**
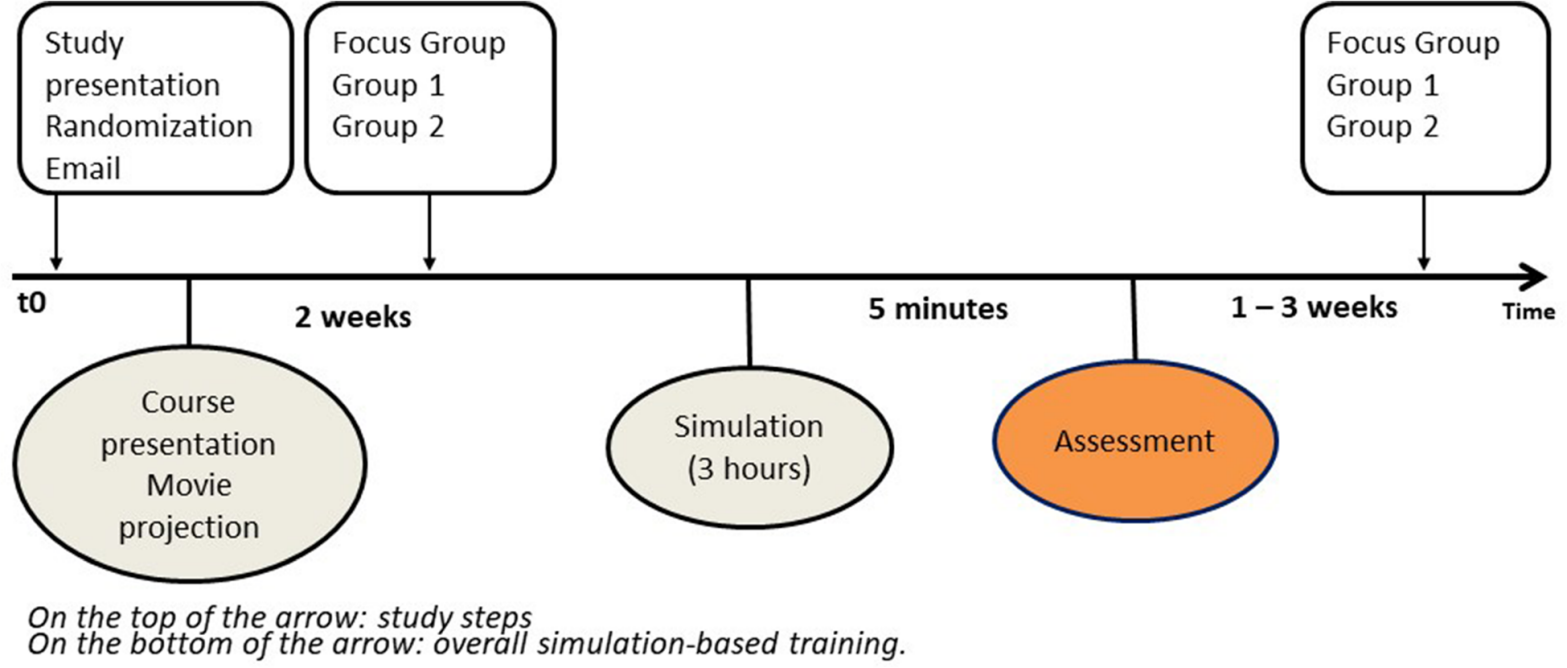
Simulation course and assessment within the Pediatric Curriculum – 5th year medical students

For both SBA (paediatrics and EM), when the medical students failed, they had to undergo another assessment session. If they failed again, they had to take the entire simulation course again.

### Participants’ selection and data collection

A total of 125 and 90 fourth- and fifth-year medical students, respectively, participated in the two curricula. There weren’t minor (< 18 years-old) students. From the overall cohort, we randomly selected forty medical students, who received an email invitation to discuss their simulation-based courses’ perceptions before and after the SBA.

Constructed by ALP and JT, the interview guide used semistructured methods with predetermined, open-ended questions. It was pilot tested with voluntary nonparticipating students to ensure that the questions were appropriate and clear (Additional file 2: Appendix 2). Before and after the SBA proceedings, the focus groups aimed to explore the medical students’ anticipation towards the SBA and to evaluate their SBA’s perceptions. The focus groups took place at the medical school or the hospital according to the participants’ preferences. ALP moderated all the focus groups as a facilitator and made field notes on relevant moments and on medical students’ attitudes. The focus groups were audio recorded, downloaded onto a computer for storage, and then transcribed verbatim. Two native English-speaking individuals, independent from the study, translated the quotes from French to English.

### Data management and analysis

Two investigators (ALP, JT) separately analysed the transcripts. With respect to the grounded theory approach, the interview transcription took place immediately after the data collection and was analysed before the next focus group using the constant comparison method [25]. After the SBA, focus groups ran on until no new themes emerged from the data. This process allowed the identification of themes and new questions or indicated theoretical saturation. The two curricula were analysed separately, as they occurred during two different periods. Focus groups were open-coded following those stages: familiarization with the transcripts with several readings identified main themes from the transcripts. We then employed open software (Iramuteq®) to perform advanced discourse analysis. The two investigators discussed the common themes before the final analysis.

## Results

Among the 215 medical students who completed the two simulation-based courses, thirty medical students (14%) participated in nine focus groups: four in the PC and five in the EM-ICM (Table 1). The focus groups had a mean duration of 71 min (+/− 12 min). The ten medical students who declined to participate in the study were either unavailable (*n* = 9) or uninterested in the study (*n* = 1). After the assessment, all the medical students passed the SBA, except for one student who failed the EM-ICM curriculum.

**Table 1:** Nine focus groups’ description

Paediatrics Curriculum			Emergency and Intensive care medicine curriculum		
	Participants (N)	Duration (min)	N° FG	Participants (N)	Duration (min)
Curriculum participants (n)	90			125	
Randomized for FG	20			20	
**Total FG participants**	**12**			**18**	
**FG Before SBA**					
*FG 1*	6	54	*FG 5*	**6**	**49**
*FG 2*	6	73	*FG 6*	**6**	**82**
**FG after SBA**					
*FG 3*	3	65	*FG 7*	**6**	**75**
*FG 4*	6	84	*FG 8*	**6**	**82**
			*FG 9*	**6**	**76**

*FG* focus group, *SBA* simulation-based assessment

After data analysis, seven themes emerged from the focus groups: four were consistently present across all focus groups (major themes); three others were not consistently present and were subject to debate (major inconsistent themes). We chose to designate them as major themes because they emerged from contradictory discussions and seemed to be important issues for the medical students. The quotations from participants are reported as follows: curriculum (PC/EM) + participant number.

### Summary of the four major themes that reached consensus (Fig. 3)

#### SBA as a support to hospital placements

Most students found that SBA would prepare them for hospital placements and be additive relative to simulation-based training alone. They emphasised the lack of feedback and supervision during their placements and great variability in the training and exposure to learning objectives. Consequently, medical students saw a motivational impact of SBA that offered meaning and a willingness to face a challenging clinical environment: *“SBA makes me want to go to work to the hospital*” *(EM 13);* “*Most of the time, I feel completely disregarded, particularly during placements, during which we spend so much time, learning so little*” *(PC2); “We are considered the insignificant medical students. We have never been shown or trained on technical procedures. For example, in my last hospital placement, we had to beg the attending physicians to show us how to use an oxygen ventimask” (EM17).*

**Fig. 3:**
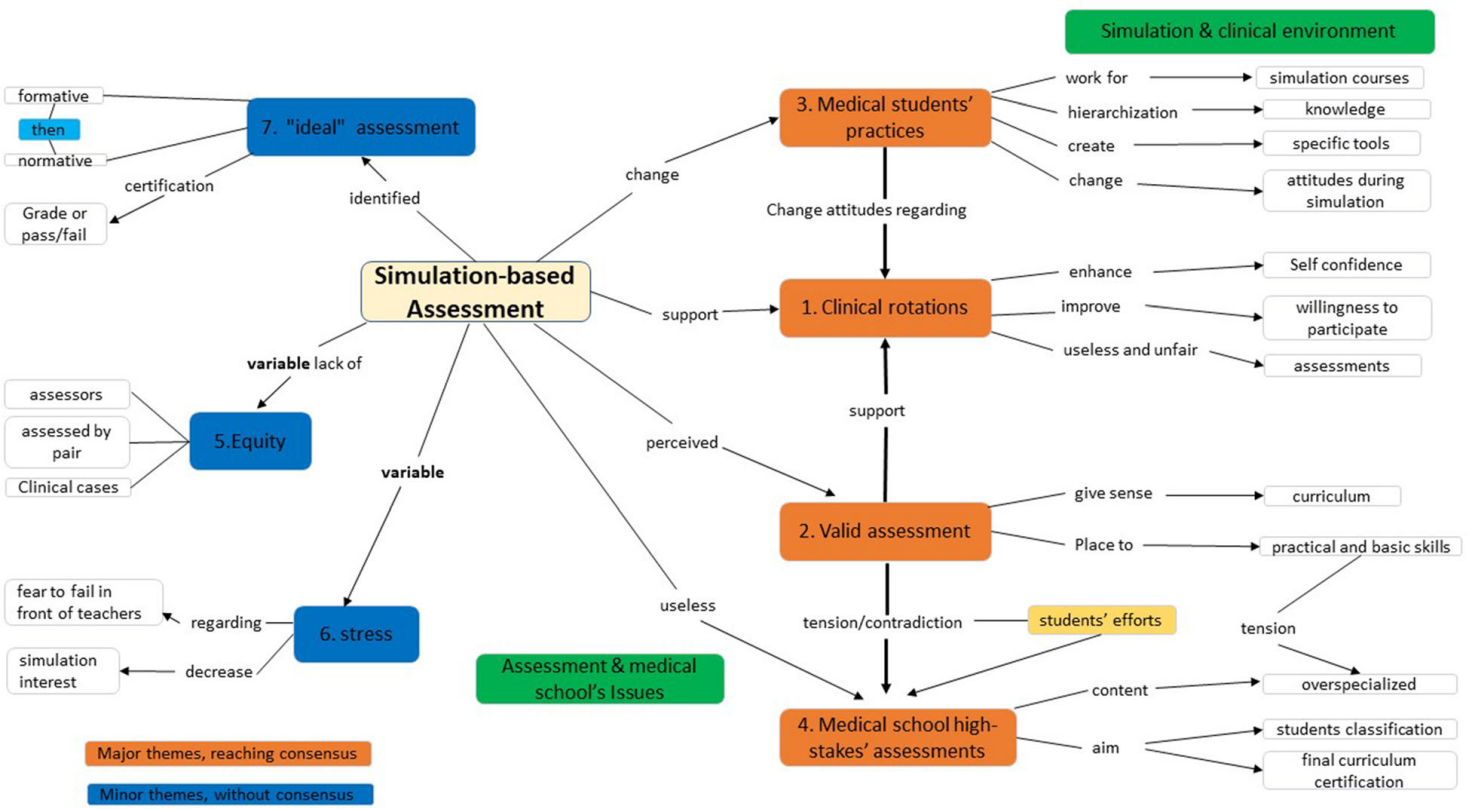
Major themes about medical students’ SBA perceptions, and their different links

Medical students perceived that SBA enhanced their self-confidence and that it would favour assertiveness within the clinical environment. The medical students felt more confident and felt able to take more initiative. They noted as follows: “*Assessment is hard, but after I succeeded, I felt like I had seen and managed the most difficult part and realised it would never be as difficult as this. Afterwards, I felt more prepared to manage the first moments of a life-threatening situation, for example” (EM 2); “SBA success could convince attending physicians to trust me and let me perform technical procedures during my hospital placements” (PC7).*

The students highlighted that SBA could be a valid tool filling a gap in assessments during placements. They described the content, tools, and organisations of the placements’ assessments as too heterogeneous, and they all pointed out the very weak validity of the final hospital placement assessments. Their main criticism was the lack of feedback on their skills. In contrast, they identified SBA as an organised assessment, probably because of the implications for medical school*: “In my final gynaecology placement assessment, I worked hard, tried to learn as much as I could, and I was asked to show the uterus on an ultrasound picture” (PC9); “As the SBA is conducted in the medical school, it’s better than placement assessments: the organisation is better, and the requirements are standardised. The training and assessment conditions can vary greatly during hospital placements […] some of my colleagues had never had training before the final oral assessment” (PC3).*

Finally, the medical students all emphasized their interest in placing practical skills at the centre of their training. After a deeper analysis of their comments, this view seemed to be associated generally with simulation-based education and not only with the existence of the assessment.

#### A major place for mannequin SBA within the overall assessment programmes

The medical students acknowledged that mannequin SBA is a tool with good face validity, and they felt that it centred practical skills in the curriculum. This view contrasted with their perception of medical school assessments, which were perceived as overly specialised and not always adapted to a competency mastery approach: *“It is a high-quality certification with a safe level for validation of skills” (PC 12); “SBA fixed the threshold very high” (PC 11); “The SBA allowed assessing ‘the basics’ although the medical school assessments often focused on the very small details” (EM5); and “it reminded us of what we must master for our future work, in contrast to knowledge that will never be useful, for example, the X mutation that leads to Y disease” (EM12).*

Some medical students also expressed doubts regarding the validity of medical school assessments and their ability to assess every competency, specifically clinical reasoning: *“It doesn’t teach how to reason about clinical conditions, it is not a smart tool” (PC7); “it is impossible to assess everything! Last year, we had a test on caregiving relationship: one of the items of the test was ‘to be empathic with the patient’ … I don’t know who didn’t select this obvious item…” (PC4).* The students found SBA to be a valid solution.

#### Mannequin SBA led to changes in students’ practices

For the medical students, SBA was a key element leading to changes. Indeed, they reported that they did not act as they would normally do before a simulation-based course. First, they all prepared for the simulation courses, while they usually attended simulation-based courses without any previous specific work: *“Assessment was like a magic world that helped me prepare all the simulation courses differently” (PC5); “assessment compelled me to revise” (PC10); and “because I knew there would be an assessment, it forced me to deepen and organise [my] theoretical knowledge and forced me to study with a different methodology” (PC2).*

Second, they described changes in their working methods: they focused on essential knowledge and tried to organise it. This illustrated the testing effect of the SBA, because of which students had to organise and mobilise their knowledge instead of only memorising it. They accomplished this with the help of specific tools that they created themselves. All students found the lack of such tools (cognitive aids or video supports) regrettable. As the students noted, *“I had never done that before, usually when I come to a simulation course or a tutorial, I just read the corresponding chapter, and not even systematically” (PC9); “SBA helped us hierarchise and organise the knowledge before the simulation courses, with a method I had never employed before” (EM 13); “I created four to six essential points for each clinical problem I could face during the simulation” (EM2); and “because we cannot find such a practical guide in our books, I had to create my own […] for each clinical case. This shows how much the books are unfit to the practice of medicine and only useful to train for the written assessments” (EM6).*

Medical students also perceived different attitudes during the simulation-based courses. Although they usually identified simulation-based training as a game and a pleasant course, they felt more focused and more involved than usual when the courses were not directed by a final summative assessment. For them, the changes were due to the need to identify the necessary skills and attitudes to pass the final exam: *“I felt more motivated to participate and, overall, more focused, as I wanted to understand what skills or knowledge would be useful for the final assessment but also for my practice during the hospital* placements*” (PC5*); *“I was involved and motivated during the training, and I pushed myself to organise my knowledge in an intelligent way” (PC4)*.

#### Simulation-based assessment is unnecessary for written high-stakes assessments

Although they considered SBA to be a useful assessment regarding their needs for hospital placements and future internships, the medical students reported that SBA was unnecessary in preparing for their final sixth-year high-stakes assessment and for their final curriculum oral and written assessments. They did not consider SBA to be helpful for success in high-stakes assessments, as it did not assess the same knowledge or skills. Throughout their medical training, they focused their efforts on succeeding in the final assessment, which will determine their professional future. Thus, they felt that one more assessment with no relationship to this goal was not essential. This view stands in opposition to the perception of the approach being helpful for hospital placements, but again, they highlighted that their future was more important: *“It doesn’t help us validate our education this year” (EM 2); “it doesn’t give us bonus points for the EM-ICM module” (EM 17); “it is just another assessment” (PC 3); and “It doesn’t assess the same knowledge as the written evaluation, so it doesn’t help us” (PC 10).*

### Summary of three other major themes inconsistent within the focus groups (Fig. 3)

#### A lack of equity

Most medical students who participated in the EM-ICM assessments reported an equity issue based on a great difference between the assessors, the scenario contents and the composition of the student pairs: “*I would have preferred another teammate; someone I knew would have been ideal. It’s not fair because my friend did not have this issue” (PEM4); “I would have preferred cardiac arrest or pneumonia over the intoxication case” (EM 14).*

The medical students perceived a disparity between the assessors and expressed some doubts on the scales’ reproducibility, grounded in their negative experience with variable content validity and reliability during the oral assessments. They also would have preferred to know the different scale contents before the final assessment session and asked for a formative assessment using the same scale rather than the summative scale. The students explained as follows: *“The scales of the oral assessments are awful, appalling, it’s a real scandal” (PC8); “For SBA, it would be useful to know the scale’s content before the assessment, it would be smart because, we are actually not aware what is expected from us. It’s disturbing, even if it is the same for the other assessment” (EM2); and “With the simulation-based assessment, we should have the opportunity to know what exactly is expected from us, like a training session with the assessment scale and adequate feedback before the final SBA” (EM16).*

#### Stressful or not?

The PC medical students did not report major stress but reported stressful moments. In contrast to medical school or hospital assessments, the preparation stage was not stressful due to the light workload: “*The simple use of the word assessment is stressful, even if I knew that I would be trained and that it would be easy” (PC 4); “Our usual assessments require five months of work, whereas for this one, which concerned only a few skills, it was easier to prepare” (PC2).*

However, the few minutes before the SBA was more stressful than the time before an oral or a written test. They described the stress of being exposed to a difficult scenario in front of the assessor. However, the students also admitted that stress was not a major issue because if they passed this test, it would help to reduce any potential stress they might feel with a real patient: “*Five minutes before the assessment, I was very, very stressed. I think it was mainly due to what the other students and teachers might think of me rather than the assessment itself. But I think this is good stress, because it exists also in real life and we have to deal with it. And if we can manage the stress here, I will probably be able to manage it in real life. I prefer to make a mistake and to feel stress with a mannequin” (PC 7).*

Conversely, half of the EM-ICM students reported stress. Even if they all recognised its benefit for clinical placements, they criticised it for exposing them to another stressful event during the difficult curriculum. The students also described losing their interest in SBT because of the stress generated by SBA: “*Although we knew the scenarios, it was stressful, more than other assessments” (EM 12); “simulation must remain a fun exercise” (EM 3); and “normally simulation-based training is nice and friendly, but with the assessment, it became stressful” (EM 9).*

#### Practical issues and nature of the assessment

The majority of the EM-ICM students and some PC students highlighted the importance of being trained before being assessed and thus to prefer formative before summative assessments. They suggested using more formative assessment tools: “*Directors’ programmes should integrate simulation earlier in the curriculum and with higher volume than currently” (PC 4)*; “*we should have more training before the assessment, just like for any other assessment, even if it means additional working time” (EM 2).*

However, the medical students all recognised the need for a summative assessment to formally recognize their abilities and performance. Afterwards, they identified formative assessments as less valuable than summative ones, probably due to misconducts considerations because they can successfully pass only by being present on the assessment day: “*I prefer to have an assessment with a real objective and with a consequence rather than an assessment without stakes” (EM10); “Tests you can easily validate by just being present are useless and unbearable” (PC 2).*

They also compared SBA with their written assessments and discussed the value of a grade for SBA. They did not reach a consensus, but some found that a grade could be helpful to identify progress and the minimal required level, although others found that the most important marker was to pass the test and to know they could use their abilities in the clinical environment. Moreover, they pointed out the main difference from the written assessment; they all appreciated the possibility of receiving feedback just after the SBA: *“We do not need a grade, what is important is to succeed, not to be perfect” (EM 13); “the grades are important in our curriculum, and they are currently used so we know how to interpret them” (PC4); and “One of the reasons for the new interest in the SBA is personal. Because it provides specific feedback, and after the session, I would exactly know what I had to work on” (EM 12).*

The last practical issue remained in the limitation of environmental fidelity, which refers to the realism of the simulation. However, this does not seem to be an obstacle for SBA, as the medical students identified many positive aspects.

### SBA highlights contradictions between two activity systems

**Fig. 4:**
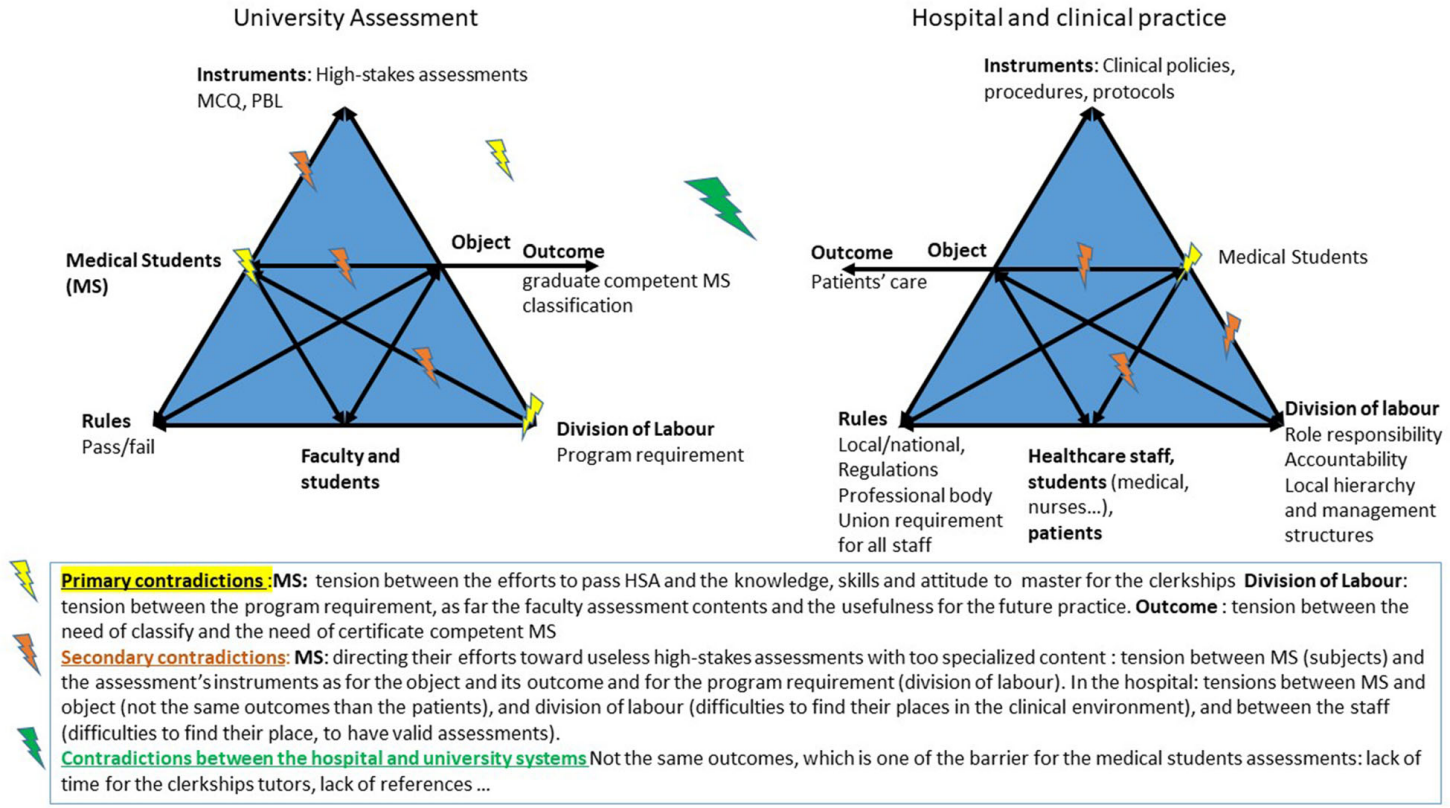
Tensions revealed by the survey illustrated by the systems of activities within which the medical students evolve

Within the grounded theory framework, the theories emerge from the data and could be analysed in relation to another theory. For this work, the relevant theoretical framework appeared to be Engeström activity theory, which focuses on the dynamics of learning and on the learner as a participant and assists in analysing the contradictions and tensions in a given system in order to help participants change [29, 30]. When debating the issue of SBA, the students constantly mentioned the challenges they faced during hospital placement. This was one of the main emergent themes, as it was not present at first during the semidirected interviews. They also made numerous comparisons between hospital placement and university training, pointing out the contradictions with an impact on their training. Engeström activity theory allowed us to analyse and illustrate these contradictions: medical students evolve in a dual system, between the university and hospital, sharing the same subjects (medical students) but with different outcomes, different rules and division of labour (Fig. 4). The hospitals’ main objectives are patient outcomes, whereas effective learning, graduation and ranking are the university outcomes. A contradiction exists between the two systems because of these different objectives. This leads to tensions for medical students, who perceive medical school as a uniform system unbiased in teaching and assessment. This is in opposition to hospital placements, where high levels of heterogeneity in the teaching and assessing methods are reported, including exposure to clinical situations.

## Discussion

Our study explored students’ perceptions of assessment with mannequin-based simulation. They underlined that SBA would be valuable in the clinical environment because it would enhance self-confidence and willingness to participate in patient management and would offer medical students’ better integration within the sometimes hostile clinical environment. The students also perceived mannequin SBA as a tool with high face validity, with the ability to centre basic skills in the curriculum and to impact their work practices. However, they found that SBA did not prepare them for the high-stakes assessments of their curriculum.

**Fig. 5:**
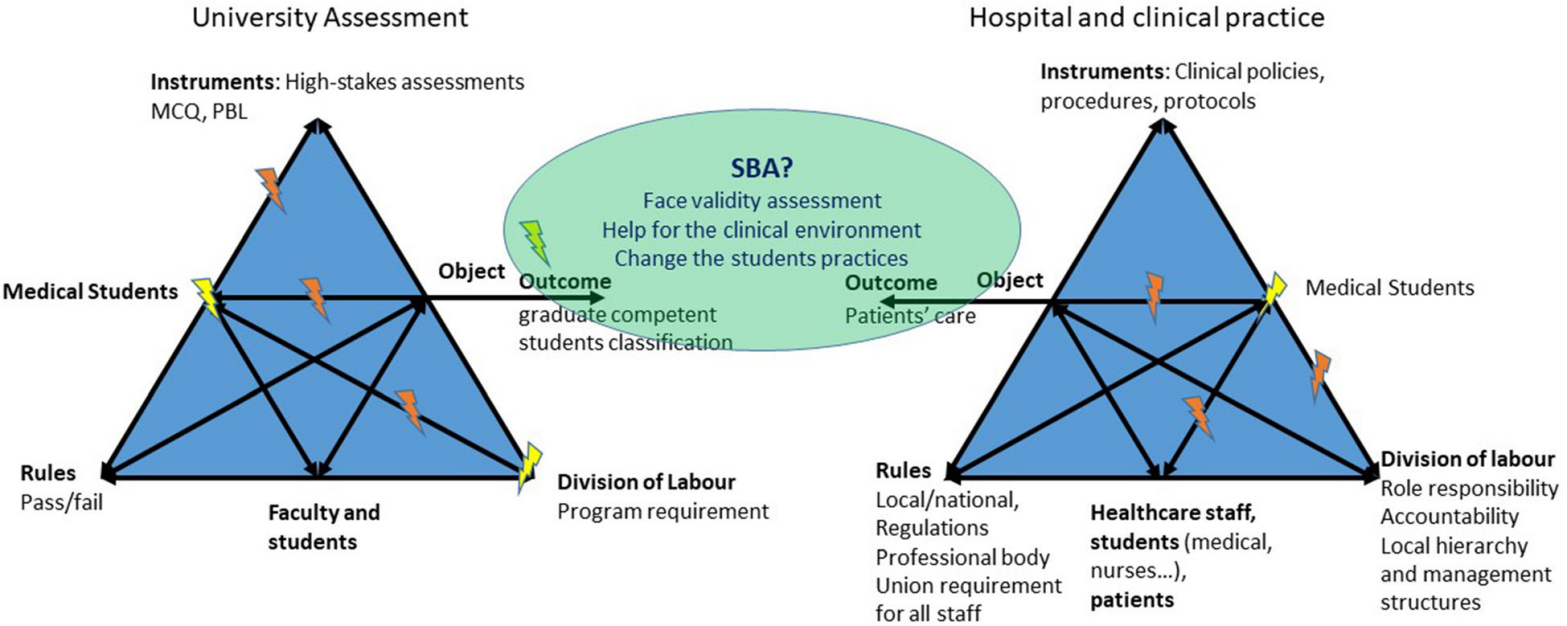
SBA’s contributions to the contradictions between the two activity systems, according to the analysis of medical students. Following the Engeström activity theory and Berragan hypothesis about the role of simulation-based training (24, 25)

The main reason for employing a posteriori the Engeström activity theory was the constant evocation of the medical students’ placement difficulties. In the data analysis, the medical students clearly stood between two systems and their specific outcomes. The Engeström activity theory has shown that expansive learning can occur when the two activity systems generate a new shared object and concept for their combined activity [30]. Berragan hypothesised that simulation-based training could be this shared object and the link between the two systems. Simulation generated a potential learning environment for medical students to practice and acquire clinical reasoning skills considering the context of the core formation systems [31]. Thus, we hypothesised that SBA could also have a specific role and place in the overall curriculum. In particular, SBA could be a tool to change not only the assessment systems but also the supervision and teaching methods used during clinical placements. Our data, focusing on SBA, suggested the same findings and emphasised Berragan’s theory (Fig. 5). Moreover, another hypothesis is that by improving their self-confidence and assertiveness within their placements, medical students could more easily develop their professional identity. Indeed, medical students’ placement is considered an experience that triggers professional identity formation, and when the students feel involved and seen as a “real doctor” by the team, it helps to construct their future professional identity [32, 33].

Another change induced by SBA was the impact on the medical students’ work routines. With this assessment, students knew the aim of the courses, and they could prepare with a certain degree of autonomy. Autonomy, motivation and control in learning are factors that enable self-regulated learning and encourage students to be active in their learning process [34]. Students have intrinsic motivation to succeed, and this is associated with deepened learning and increased control of their own outcomes, which could decrease feelings of distress [35].

The students were worried about their final high-stakes assessment and reported that SBA was not an efficient tool to prepare them for this very important step in their curriculum. This observation indicates another contradiction and tension within their curriculum. As described above, the final year assessment focuses on knowledge assessment, which is more often overly specialised and unaligned with the SBA content. This issue underlines the need to align the different teaching and assessment tools. Such alignment is lacking between SBA and the final-year written assessment [36], and our results highlight the impact of a lack of alignment on students’ motivation to participate in learning activities. Thus, our findings support the need for future reform to emphasise the place of SBA in the curriculum.

When the students identified SBA as an unfair assessment, they mentioned the subjectivity and lack of authenticity. However, subjectivity is one of the inherent pitfalls of a competency-based assessment [37]. The nature of competency is a multicomponent object, with exteriorised and measurable performance but also hidden components such as mobilisation of internal resources or clinical reasoning. Our hypothesis is that medical students rejected the subjectivity because it is not aligned with “students’ culture” [38]. Indeed, in past decades, a valid assessment tool was defined as quantitative and objective. The challenges for faculty are to understand and deal with this subjectivity to create new assessment frameworks different from MCQ [7]. This will help educators and students to employ less objective assessments, such as multimodal ones with several tools and situations in a whole programmatic assessment [39]. Simulation has a great role to play because it employs controlled reproducible, reliable environments with controlled subjectivity. It is the main aim of OSCEs, but even if they are standardised simulation assessments, they reflect student performance and have good validity and reliability only if they use different contexts with different raters: at least 8 stations from five to 10 minutes [7, 40, 41]. Teachers and medical schools must pay attention to these issues, which seem to be important to many medical students. For this reason, the recent Canadian recommendations emphasise the need to maintain simulation courses as a “safe place”. The use of formative assessments should remain a major place for explicit feedback. They also underline the current need for standardisation with reproductive, valid and reliable assessments. The use of respected professional activities would likely be seen as helpful [42].

Discussions on the practical aspects of the SBA highlighted two major characteristics of such an assessment: feedback and ratings. Normative assessment does not always provide feedback, especially in the French context, where feedback is provided through grades or classifications without qualitative feedback. With systematic debriefings, SBA could provide students with accurate feedback. However, grades were not completely approved by the students. They preferred knowing that they had the required skills without rankings [43]. Some authors also found these results, with a specific element: the more they progressed in the curriculum, the more they felt demotivation towards the ranking system [44]. Grades engage extrinsic motivation, which is linked to short-term memory and surface learning, unlike intrinsic motivation, which aids in self-development, satisfaction with an accomplished task and increased efficacy [35]. One other suggestion based on our results is the ethical issue of our assessment. As previously shown, debriefing is an essential part of simulation-based training [45, 46]. During the assessment session, the debriefing was shorter than that during the training courses. However, it was appreciated by the students because it was the first time they were provided with individual feedback immediately after an assessment. For simulation practice, it could be viewed as a short, weak debriefing and could contribute to the perception of unfairness of SBA. A possible improvement would be to give each student personal feedback with individual improvement goals [47].

Another ethical concern is the stress linked to the assessment process. Simulation-based training is supposed to be a safe environment to learn with opportunities to make errors and learn from these errors. However, this training environment has been shown to be stressful [48]. If we add stress to assessments, it could deflect SBT from one of its important aims: safe learning.

For these different reasons, caution should be applied in SBA for medical students, and we should improve our simulation tools and environments.

### Limits

This study presents some limitations. First, it is a single-centre study, but two different SBAs took place, and thirty students did not have the same clinical experiences. The variety of experiences contribute to the authenticity of the study. Moreover, we obtained data saturation with the eighth focus group. Second, we missed a step in the qualitative approach, as we did not send back the findings to the participants. This would have improved the validity of the study by ensuring that the participants’ ideas were accurately represented. Third, the medical students’ perception highlighted the tension to which they are exposed within the two activity systems. It would have been helpful to complete the data by including observations of their activity within the simulation-based courses, assessments and placement. Moreover, even if it was not our main objective, we could have obtained insight from teachers to obtain more complete information regarding the medical students’ perceptions.

## Conclusion

Medical students’ perceived SBA was a valid assessment tool with the capacity to enhance their self-confidence and willingness to participate in hospital placements. The assessment provided them the necessary opportunity to learn to take care of patients safely. They found that the experience had a positive impact on their practices; however, they regretted that it could be unfair, stressful and useless for their final high-stakes written assessment. The data analysis highlighted the several contradictions that medical students face within their two training systems. It was a relevant hypothesis that SBA could be an interesting link between these two systems, with a dedicated role to play in addressing the challenges faced by medical students between hospital placements and medical school requirements. These results are inspiring and should lead to the improvement and development of simulation-based assessments throughout medical school curricula. Our results describe a preimplementation mannequin SBA and OSCE and provide valuable insight for programme designers.

## Supplementary Information

**Additional file 1: Appendix 1**. EM-ICMC’s scenarios and focus groups.

**Additional file 2: Appendix 2**. Example of focus groups within the paediatric curriculum.

**Additional file 3: Annex 1**. Pediatrics’ Basic Life Support : Score used for the simulation-based assessment.

## Data Availability

Data supporting the results are available in a safe, secured electronic file on ALP’s professional computer. All the transcripts are available if needed. Therefore, data are available from the corresponding author upon reasonable request.

## Abbreviations

CBME: Competency-based medical education
EM-ICMC: Emergency and intensive care medicine curriculum
EM1: Student one from EM-ICMC
OSCE: Objective structured clinical examination
PC: Paediatric curriculum
PC1: Student one from PC
SBA: Simulation-based assessment
SBT: Simulation-based training
SRLF: French Society of Intensive Care Medicine

## References

[1] Batalden P, Leach D, Swing S, Dreyfus H, Dreyfus S (2002). General competencies and accreditation in graduate medical education. Health Aff (Millwood).

[2] Englander R, Carraccio C (2018). A Lack of Continuity in Education, Training, and Practice Violates the “Do No Harm” Principle. Acad Med.

[3] Carraccio CL, Englander R (2013). From Flexner to competencies: reflections on a decade and the journey ahead. Acad Med.

[4] Carraccio C, Englander R, Holmboe ES, Kogan JR (2016). Driving care quality: aligning trainee assessment and supervision through practical application of Entrustable professional activities, competencies, and milestones. Acad Med.

[5] Ten Cate O, Chen HC, Hoff RG, Peters H, Bok H, van der Schaaf M (2015). Curriculum development for the workplace using Entrustable professional activities (EPAs): AMEE guide no. 99. Med Teach.

[6] Lurie SJ, Mooney CJ, Lyness JM (2009). Measurement of the General Competencies of the Accreditation Council for Graduate Medical Education: A Systematic Review. Acad Med.

[7] Epstein RM (2007). Assessment in medical education. N Engl J Med.

[8] Holmboe ES, Ward DS, Reznick RK, Katsufrakis PJ, Leslie KM, Patel VL (2011). Faculty Development in Assessment: The Missing Link in Competency-Based Medical Education. Acad Med.

[9] Govaerts MJB, van der Vleuten CPM, Schuwirth LWT, Muijtjens AMM (2007). Broadening perspectives on clinical performance assessment: rethinking the nature of in-training assessment. Adv Health Sci Educ Theory Pract mai.

[10] Beeson MS, Vozenilek JA (2014). Specialty milestones and the next accreditation system: an opportunity for the simulation community. Simul Healthc.

[11] Bennett RE (2011). Formative assessment: a critical review. Assess Educ.

[12] Griswold S, Fralliccardi A, Boulet J, Moadel T, Franzen D, Auerbach M, Hart D, Goswami V, Hui J, Gordon JA (2018). Simulation-based education to ensure provider competency within the health care system. Acad Emerg Med.

[13] Van Der Vleuten CPM (1996). The assessment of professional competence: developments, research and practical implications. Adv Health Sci Educ.

[14] Ahmed K, Jawad M, Dasgupta P, Darzi A, Athanasiou T, Khan MS (2010). Assessment and maintenance of competence in urology. Nat Rev Urol.

[15] Doughty CB, Kessler DO, Zuckerbraun NS, Stone KP, Reid JR, Kennedy CS, Nypaver MM, Auerbach MA (2015). Simulation in pediatric emergency medicine fellowships. Pediatrics..

[16] McMurray L, Hall AK, Rich J, Merchant S, Chaplin T (2017). The nightmares course: a longitudinal, multidisciplinary, simulation-based curriculum to train and assess resident competence in resuscitation. J Grad Med Educ.

[17] Langdon MG, Cunningham AJ (2007). High-fidelity simulation in post-graduate training and assessment: an Irish perspective. Ir J Med Sci.

[18] Chaplin T, Thoma B, Petrosoniak A, Caners K, McColl T, Forristal C (2020). Simulation-based research in emergency medicine in Canada: Priorities and perspectives. CJEM.

[19] Cook DA, Zendejas B, Hamstra SJ, Hatala R, Brydges R (2014). What counts as validity evidence? Examples and prevalence in a systematic review of simulation-based assessment. Adv Health Sci Educ Theory Pract..

[20] Cook DA, Brydges R, Ginsburg S, Hatala R (2015). A contemporary approach to validity arguments: a practical guide to Kane’s framework. Med Educ.

[21] Watling CJ, Kenyon CF, Schulz V, Goldszmidt MA, Zibrowski E, Lingard L (2010). An exploration of faculty perspectives on the in-training evaluation of residents. Acad Med.

[22] Cilliers FJ, Schuwirth LWT, Herman N, Adendorff HJ, van der Vleuten CPM (2012). A model of the pre-assessment learning effects of summative assessment in medical education. Adv Health Sci Educ Theory Pract.

[23] Tong A, Sainsbury P, Craig J (2007). Consolidated criteria for reporting qualitative research (COREQ): a 32-item checklist for interviews and focus groups. Int J Qual Health Care.

[24] Corbin J, Strauss A (2008). Basics of Qualitative Research (3rd ed.): Techniques and Procedures for Developing Grounded Theory.

[25] Lingard L, Albert M, Levinson W (2008). Grounded theory, mixed methods, and action research. BMJ..

[26] Kennedy TJT, Lingard LA (2006). Making sense of grounded theory in medical education. Med Educ.

[27] Stalmeijer RE, Mcnaughton N, Van Mook WNKA (2014). Using focus groups in medical education research: AMEE guide no. 91. Med Teach..

[28] Maconochie IK (2015). Bingham R, Eich C, López-Herce J, Rodríguez-Núñez A, Rajka T, et al. European resuscitation council guidelines for resuscitation 2015. Resuscitation.

[29] Engestrom Y (2000). Activity theory as a framework for analyzing and redesigning work. Ergonomics.

[30] Engeström Y (2001). Expansive learning at work: toward an activity theoretical reconceptualization. J Educ Work.

[31] Berragan L (2013). Conceptualising learning through simulation: an expansive approach for professional and personal learning. Nurse Educ Pract.

[32] Maitra A, Lin S, Rydel TA, Schillinger E (2021). Balancing forces: medical students’ reflections on professionalism challenges and professional identity formation. Fam Med.

[33] Kay D, Berry A, Coles NA (2019). What experiences in medical school trigger professional identity development?. Teach Learn Med.

[34] White CB (2007). Smoothing out transitions: how pedagogy influences medical students’ achievement of self-regulated learning goals. Adv Health Sci Educ Theory Pract.

[35] Pelaccia T, Viau R (2017). Motivation in medical education. Med Teach..

[36] Schuwirth LWT, van der Vleuten CPM (2011). General overview of the theories used in assessment: AMEE guide no. 57. Med Teach.

[37] Van der Vleuten CP, Norman GR, De Graaff E (1991). Pitfalls in the pursuit of objectivity: issues of reliability. Med Educ.

[38] Laqueur T (2002). Boys in White: Student Culture in Medical School. BMJ.

[39] Schuwirth LWT, van der Vleuten CPM (2012). Programmatic assessment and Kane’s validity perspective. Med Educ.

[40] Khan KZ, Gaunt K, Ramachandran S, Pushkar P (2013). The objective structured clinical examination (OSCE): AMEE guide no. 81. Part II: Organisation & Administration. Med Teach..

[41] Khan KZ, Ramachandran S, Gaunt K, Pushkar P (2013). The objective structured clinical examination (OSCE): AMEE guide no. 81. Part I: an historical and theoretical perspective. Med Teach.

[42] Hall AK, Chaplin T, McColl T, Petrosoniak A, Caners K, Rocca N, Gardner C, Bhanji F, Woods R (2020). Harnessing the power of simulation for assessment: consensus recommendations for the use of simulation-based assessment in emergency medicine. CJEM.

[43] Rohe DE, Barrier PA, Clark MM, Cook DA, Vickers KS, Decker PA (2006). The benefits of pass-fail grading on stress, mood, and group cohesion in medical students. Mayo Clin Proc.

[44] O’Neill P, Baxter CM, Morris J (1999). Does awarding a medical degree with honours act as a motivator or demotivator to student learning?. Med Educ.

[45] Issenberg SB, McGaghie WC, Petrusa ER, Lee Gordon D, Scalese RJ (2005). Features and uses of high-fidelity medical simulations that lead to effective learning: a BEME systematic review. Med Teach.

[46] Dieckmann P, Molin Friis S, Lippert A, Ostergaard D (2009). The art and science of debriefing in simulation: ideal and practice. Med Teach.

[47] Motola I, Devine LA, Chung HS, Sullivan JE, Issenberg SB (2013). Simulation in healthcare education: a best evidence practical guide. AMEE guide no. 82. Med Teach.

[48] Bong CL, Lightdale JR, Fredette ME, Weinstock P (2010). Effects of simulation versus traditional tutorial-based training on physiologic stress levels among clinicians: a pilot study. Simul Healthc.

