# Supplementary material for: Medical students’ perception of simulation-based assessment in emergency and paediatric medicine: a focus group study
Source: BMC Med Educ. 2021 Nov 19;21:586. doi: 10.1186/s12909-021-02957-5 (PMC8605506; doi:10.1186/s12909-021-02957-5)
Supplement: Supplementary file 1 — Additional file 1: Appendix 1. EM-ICMC’s scenarios and focus groups. [file 12909_2021_2957_MOESM1_ESM.docx]

Appendix 1: EM-ICMC’s scenarios and focus groups

**A. list of the 12 EM-ICMC’s scenarios**

1. Acute severe asthma
2. Head trauma under Vitamin-K Antagonist
3. Hyperkalaemia and bradycardia
4. Acute left heart failure
5. Sepsis and pneumonia
6. Cardiac arrest and ventricular fibrillation
7. Cardiac arrest and asystolia
8. Cardiac arrest and pulseless electrical activity
9. Anaphylactic shock
10. Digestive haemorrhage
11. Voluntary intoxication with tricyclic agents
12. Acute coronary syndrome, ST elevation +

**B. Example of focus group’s content before simulation-based assessment session**

**Introduction:** for the respondents, the introduction is the time where they took place around the table, told their name, where they made their hospital clerkship and to rest. The aim is to relax, and to know more about the study.

1. The interviewer presented herself as a learning sciences student in mastered degree and asked their first names to the participants. She thanked the students to be here.
2. The she presented the research and explained the research process:

- Qualitative study and its particularity
- The interest of focus groups: to discus, to give their different points of view
- There is no wrong answer
- The report is anonymous for the other researchers, and for the faculty. Nobody in he faculty is aware about your participation

1. After all, she presented the aim of the research:

To learn the effects of simulation-based assessment

To learn the effects of simulation-based training

1. Then, she showed them the material used to audio-record the focus group.
2. At the end, they signed an approval for their participation in the study

**Interview**: there were opened questions

1. What does the simulation-based assessment represent for you?
2. Tell us one of your experiences with SBT
3. About the assessment in medicine curricula: what are you thinking about the different ways to assess you?
4. How do you imagine an assessment with simulation?
5. Next week, you will be assessing on a manikin, for the first time. What do you think about it? How do you feel?
6. How do you prepare your EM-ICM lectures?
7. How did you prepare your simulations courses?
8. How do you imagine the medical students’ certification in the future?

**C. Example of focus group’s content after simulation-based assessment session, with or without normative assessment**

**Introduction:** for the respondents, the introduction is the time where they took place around the table, told there name, where they made their hospital clerkship and to rest. The aim is to relax, and to know more about the study.

1. The interviewer presented herself again as a learning sciences student in mastered degree and asked their first names to the participants. She thanked the students to be here.
2. she presented the research and explained the research process:

- Qualitative study and its particularity
- The interest of focus groups: to discus, to give their different points of view
- There is no wrong answer
- The report is anonymous for the other researchers, and for the faculty. Nobody in the faculty is aware about your participation

1. After all, she presented the aim of the research:

To learn the effects of simulation-based assessment

To explore the effects of simulation-based training

1. Then, she showed them the material used to audio-record the focus group.
2. At the end, they signed an approval for their participation in the study

**Interview**: there were opened questions

1. Did you prepare the assessment, and if yes, how?
2. Did you feel different before the session? In comparison with the two others SBT courses? In comparison with a lecture? in comparison with the other EM-ICM module evaluations?
3. Can you share your opinion: from the beginning to the end of the session?
4. How did you feel?
5. How did you approach the simulation?
6. How do you imagine the completion of such an exercise?
7. How did you feel in a binomial team?
8. How do you see this assessment included in your curriculum? (if no answer, to help) Which place?
9. To your opinion, what could be the role of the simulation-based assessment?
10. To your opinion, how are the global assessments in medical school?
11. For you, what is the main role of an assessment?
