# Supplementary figures and images for: Medical students’ perception of simulation-based assessment in emergency and paediatric medicine: a focus group study
Source: BMC Med Educ. 2021 Nov 19;21:586. doi: 10.1186/s12909-021-02957-5 (PMC8605506; doi:10.1186/s12909-021-02957-5)

**Annex 1: Pediatrics’ Basic Life Support : Score used for the simulation-based assessment.**


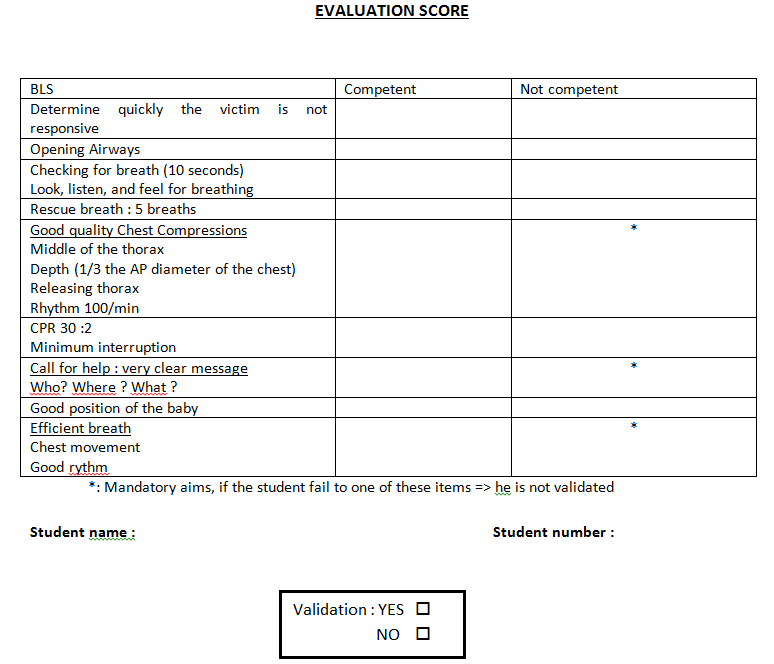

Supplement: Supplementary file 3 — Additional file 3: Annex 1. Pediatrics’ Basic Life Support : Score used for the simulation-based assessment. [file 12909_2021_2957_MOESM3_ESM.docx]
